# Editorial: Highlights in Translational Research in Rehabilitation 2021/22

**DOI:** 10.3389/fresc.2023.1200547

**Published:** 2023-05-02

**Authors:** Ada W. S. Leung, Behdin Nowrouzi-Kia

**Affiliations:** ^1^ Department of Occupational Therapy, Faculty of Rehabilitation Medicine, College of Health Sciences, University of Alberta, Edmonton, AB, Canada; ^2^ Neuroscience and Mental Health Institute, University of Alberta, Edmonton, AB, Canada; ^3^ Department of Occupational Science and Occupational Therapy, University of Toronto, Toronto, Ontario, Canada

**Keywords:** translational research, rehabilitation, knowledge transfer, knowledge translation, intervention

**Editorial on the Research Topic** Highlights in translational research in rehabilitation 2021/22

Translational research encompasses a wide spectrum of concepts, depending on the translation's discipline and focus. From a biological perspective, translational research concerns the translation of knowledge from basic science research into clinical medicine. As Woolf (1) has mentioned, this area of research, the interface between basic science and clinical medicine, is geared towards generating new treatments, such as drugs and invasive procedures, that can be applied clinically and commercially. This area of translational research requires the establishment of a variety of knowledge from genetics and molecular biology, as well as supportive technologies to bring basic science to clinical practice. Thus, the research outcome fosters new techniques and approaches for preventing, diagnosing, and treating diseases necessary for maintaining or improving health (2). From a socio-economical perspective, translation research ensures that the new treatment techniques and approaches reach the people or patients who need them. This area of research typically involves knowledge users, such as healthcare providers, researchers, patients, and the public, an integrative process of knowledge transfer, and methodologies for outcome evaluation. Thus, this area of translational research concerns not only the treatment techniques that are to be translated into clinical practice but also the quality of treatment, such as the patient-clinician relationship, the quality of care, the ease of service access, etc. In other words, the research fosters the multidirectional and multidisciplinary integration of basic research, patient-oriented research, and population-based research, with the long-term aim of improving the public's health (3). In short, regardless of the nature of knowledge transfer, whether biological or socio-economical, the goal of translational research is to close the gap between new scientific discoveries and their endpoint application to clinical practice, interventions, decision-making, and health policy (4, 5).

Rehabilitative interventions are often longitudinal in nature and are implemented through therapeutic interaction in real-life situations as well as virtual reality environments to promote functional recovery. For a new intervention or approach to be successfully translated into clinical practice, the effectiveness of the intervention or approach must first be established through rigorous experimental and/or clinical testing, followed by the implementation and evaluation of the effective intervention and assessment procedures in clinical practice. Thus, the focus of translational research is diverse, covering different aspects as mentioned above. The current research topic highlights four unique studies that have emphasized translational research in clinical practice. Their research spans different focuses, including assessing the design of assistive devices, testing longitudinally throughout a recovery process, optimizing the administrative process of an assessment tool, and evaluating a strategy to facilitate the delivery of health services.

Aranceta-Garza and Ross examined the limitation of current designs of prefabricated wrist-hand orthoses (WHOs). Their study raised questions that challenge the design of the WHOs though they are widely used clinically. The study highlighted the gap between the scientific design of the assistive device and its safe and effective applications for clinical practice. This was an important area in the field of health science. The functionality of an assistive device could not be maximized without fitting its purpose defined by the users. Thus, the study is one of the important areas in translational research.

Vuong et al. focused on examining the longitudinal change of patients who were recovering from traumatic brain injury (TBI). Their study tested cognitive abilities and gait variables along the recovery trajectory, recognizing that both cognition and physical components contribute to functional performance. This study highlighted the importance of tracking different elements in the recovery process and examining the inter-relation of the elements throughout functional recovery. Additionally, the consideration of cognitive and physical outcomes and their relationships in the recovery process appears to be beneficial to the development of rehabilitation approaches for people with TBI.

Alfaro et al. examined the administrative process of the InterRAI Community Health Assessment, a tool used for evaluating older adults’ vision and hearing loss. This kind of translational research assists in optimizing the assessment process and generating recommendations for improving the evaluation process in clinical practice. The results could help inform the efficiency of clinical practice.

Harding et al. used mixed methods to evaluate a strategy—the Specific Timely Appointments for Triage (STAT)—that could be implemented to reduce healthcare waiting time. The evidence obtained was translated into clinical practice to improve access to healthcare. This could set an example for future studies that aim to demonstrate the uptake of evidence to improve the delivery of health services.

In summary, translational research is an essential part of evidence-based clinical practice. It connects knowledge, scientists, inventors, engineers, healthcare professionals, patients, users, administrators, and policymakers together to deliver quality care within the healthcare system. The studies presented in this research topic offer readers some insights and references into the scope of translational research.
